# Isolated nonparathyroid hormone-mediated hypercalcemia: a rare presentation of retroperitoneal sarcoidosis

**DOI:** 10.1210/jcemcr/luag028

**Published:** 2026-02-25

**Authors:** Chao Xue, Ranjini Vengilote, Astrid Aviles-Melendez, Alshaima Yousef, Michael Quartuccio

**Affiliations:** Department of Internal Medicine, Rochester General Hospital, New York, NY 14621, USA; Department of Internal Medicine, Rochester General Hospital, New York, NY 14621, USA; Department of Endocrinology, Diabetes and Metabolism, Rochester General Hospital, New York, NY 14607, USA; Department of Internal Medicine, Rochester General Hospital, New York, NY 14621, USA; Department of Endocrinology, Diabetes and Metabolism, Rochester General Hospital, New York, NY 14607, USA

**Keywords:** hypercalcemia, parathyroid hormone, sarcoidosis, retroperitoneal

## Abstract

Non-parathyroid hormone (PTH)-mediated hypercalcemia has diverse etiologies, including granulomatous disorders such as sarcoidosis, in which extrarenal 1-α-hydroxylase activity leads to excess production of 1,25-dihydroxyvitamin D (1,25(OH)₂D). We report a rare case of sarcoidosis presenting as isolated hypercalcemia without pulmonary involvement, complicated by initially normal 1,25(OH)₂D levels and a false-negative core biopsy. A 44-year-old man with hypertension and type 2 diabetes presented with malaise, polyuria, and polydipsia. Laboratory evaluation showed severe hypercalcemia with suppressed PTH. PTH-related peptide, 25-hydroxyvitamin D, TSH, and albumin were normal. Imaging revealed no osseous lesions or lymphadenopathy. He improved with intravenous fluids, calcitonin, and zoledronic acid. Three months later, he re-presented with recurrent hypercalcemia and elevated 24-hour urinary calcium excretion. Outpatient evaluation revealed elevated 1,25(OH)₂D and retroperitoneal lymphadenopathy. Core needle biopsy was nondiagnostic, but subsequent excisional biopsies of retroperitoneal lymph node confirmed nonnecrotizing granulomatous inflammation. He was diagnosed with sarcoidosis and started on high-dose steroids, later transitioning to mycophenolate. With that treatment, calcium levels normalized quickly. This case highlights the diagnostic complexities of non-PTH-mediated hypercalcemia and underscores the importance of a comprehensive workup, including medication review, laboratory tests, radiography, and biopsy, with consideration for excisional biopsy.

## Introduction

Non-parathyroid hormone (PTH)-mediated hypercalcemia includes a broad set of etiologies, most commonly caused by hypercalcemia of malignancy. However, calcitriol overproduction from either malignancy or granulomatous processes is a possibility. Sarcoidosis is a known etiology of hypercalcemia, typically presenting with lung lesions, though rarely presenting without pulmonary manifestations. Here, we report a case of nonpulmonary sarcoidosis presenting with severe, non-PTH-mediated hypercalcemia.

## Case presentation

A 44-year-old man with a history of hypertension and type 2 diabetes presented with malaise, polyuria, and polydipsia for 2 weeks.

## Diagnostic assessment

His laboratory results revealed severe hypercalcemia, with serum calcium of 16.4 mg/dL (SI: 4.09 mmol/L) (reference range: 8.3-10.6 mg/dL; 2.1-2.6 mmol/L) and a low PTH level of 7.3 pg/mL (SI: 0.77 pmol/L) (reference range: 18-88 pg/mL; 1.6-6.9 pmol/L). PTH-related peptide (PTHrP) (0.4 pmol/L, reference range: < 4.2 pmol/L), 25-hydroxy vitamin D (25(OH)D) (22 ng/mL; SI: 55 nmol/L) (reference range: 20-50 ng/mL; 50-125 nmol/L), 1,25-dihydroxyvitamin D (1,25(OH)_2_D) (42 pg/mL; SI: 104.8 pmol/L) (reference range: 18-64 pg/mL; 45-160 pmol/L), TSH (2.95 µIU/mL; SI: 2.95 mIU/L) (reference range: 0.55-4.78 µIU/mL; 0.5-5.0 mIU/L), and albumin levels (4.3 g/dL; SI: 43 g/L) (reference range: 3.2-4.8 g/dL; 32-48 g/L) were all within normal limits. His vitamin A level was elevated at 112 µg/dL (SI: 3.91 µmol/L) (reference range: 32.5-78 µg/dL; 1.13-2.72 µmol/L). Bone survey showed no lytic or destructive osseous lesions, and chest X-ray revealed no signs of hilar or mediastinal lymphadenopathy. He denied using thiazide diuretics or vitamin A supplements.

Treatment led to a partial improvement in calcium levels; however, the etiology of hypercalcemia remained unclear at the time of discharge, as further laboratory tests and imaging studies were still planned. He was advised to follow up with nephrology and endocrinology for additional diagnostic evaluation.

Three months later, he returned with similar symptoms and an elevated calcium level of 14.3 mg/dL, again with suppressed PTH. PTHrP, (25(OH)D), and angiotensin-converting enzyme (ACE) levels were normal. His 24-hour urine calcium was elevated at 719 mg (SI: 17.9 mmol/24 hours) (reference range: 100-300 mg/24 hours; 2.5-7.5 mmol/24 hours).

**Figure 1:**
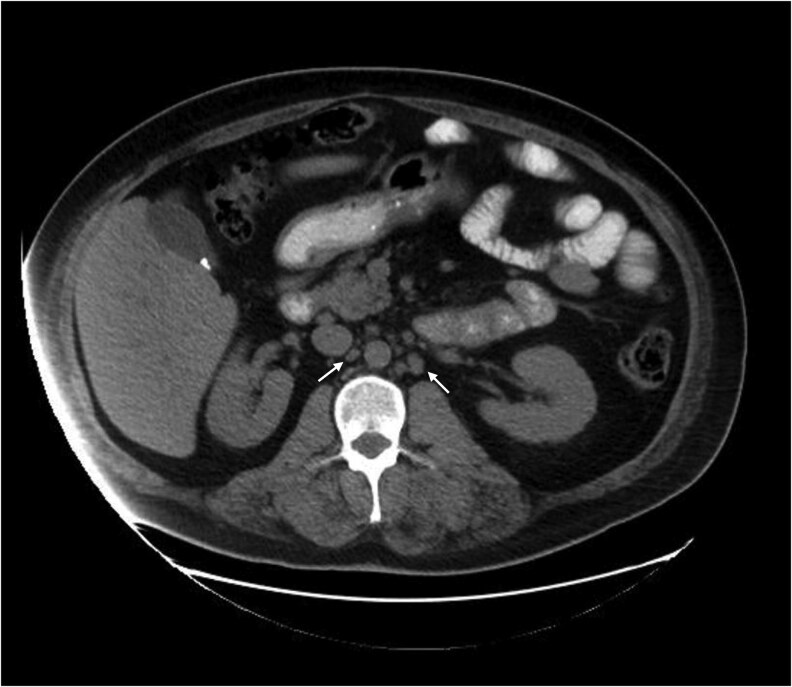
CT abdomen scan revealed mildly prominent retroperitoneal, aortocaval, and para-aortic lymph nodes (arrows).

**Figure 2:**
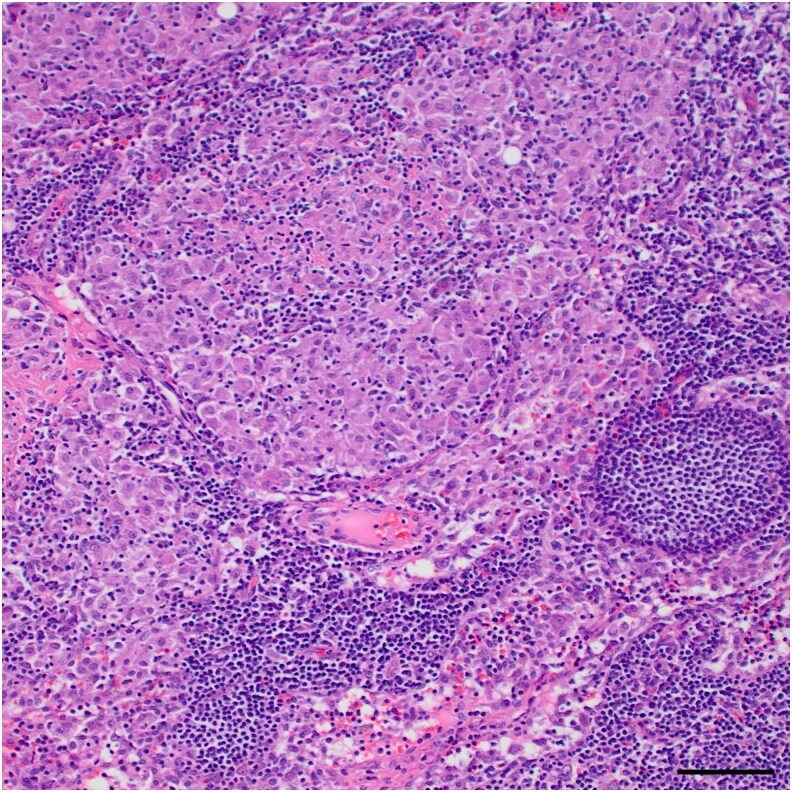
Excisional biopsy of retroperitoneal lymph node revealed nonnecrotizing granulomatous inflammation (hematoxylin and eosin, 10×, scale bar = 200 μm).

During outpatient follow-up with endocrinology, his 1,25(OH)_2_D levels were found to be elevated at 84 pg/mL (201 pmol/L). Computed tomography (CT) scan did not show pulmonary atypia but revealed retroperitoneal lymphadenopathy (Fig. 1). He underwent a core needle biopsy of the retroperitoneal lymph node, which showed benign lymphoid tissue without malignancy. Subsequently, he had excisional biopsies of the liver, peritoneum, and retroperitoneal lymph node, which revealed nonnecrotizing granulomatous inflammation (Fig. 2), consistent with sarcoidosis.

## Treatment

During the first hospitalization, he was treated with fluids, received 4 doses of 400 units of calcitonin and a single 4-mg dose of zoledronic acid, resulting in an improvement of serum calcium to 11.6 mg/dL before discharge. During the second hospitalization, he was again treated with fluids and 4 doses of 400 units of calcitonin, which reduced his serum calcium level to 10.8 mg/dL. He was discharged on cinacalcet 30 mg, taken 3 times per week.

Following histopathologic confirmation of sarcoidosis, he was started on prednisone 30 mg daily, later transitioning to mycophenolate 1000 mg twice daily.

## Outcome and follow-up

Steroid and mycophenolate rapidly normalized his hypercalcemia, which has persisted.

## Discussion

Mechanistically, non-PTH-mediated hypercalcemia can occur through 3 mechanisms: increased intestinal calcium absorption, enhanced renal calcium reabsorption, and increased bone resorption. Intestinal absorption increases with elevated 1,25(OH)_2_D in the setting of excess intake or increased production of 1,25(OH)_2_D in granulomatous diseases, such as sarcoidosis. Renal reabsorption can be augmented by PTHrP, vitamin D, thiazide diuretics, or volume depletion. Bone resorption is driven by osteoclast activation, often mediated by malignancy-derived PTHrP (either produced locally or ectopic), vitamin A or D excess, thyrotoxicosis, adrenal insufficiency, or direct bone destruction by malignancy [1-4].

For non-PTH-mediated hypercalcemia, evaluation involves measuring serum PTHrP levels and vitamin D metabolites such as 25(OH)D and 1,25(OH)_2_D [5-7]. Elevated PTHrP is seen in malignancy, whereas elevated 1,25(OH)_2_D can be seen with vitamin D intoxication or enhanced extrarenal 1-α-hydroxylase activity, often in granulomatous diseases or lymphoma [1-3]. Of note, 1,25(OH)_2_D levels may appear normal but still inappropriately high in the setting of hypercalcemia. In our case, the initial 1,25(OH)_2_D level was within the normal range; however, following treatment with calcitonin and zoledronic acid, the reduction of serum calcium level eventually unmasked the elevated 1,25(OH)_2_D level during outpatient follow-up. In the setting of elevated 1,25(OH)_2_D, further workup includes imaging studies such as chest radiograph. If negative, chest, abdominal, and pelvic CT scans can identify pulmonary abnormalities and/or lymphadenopathy commonly associated with granulomatous disease and lymphoma [8-10]. Elevated ACE levels can support the diagnosis of sarcoidosis, although it is not specific [11]. Chitotriosidase is produced by activated macrophages, and several studies have suggested its utility as a reliable biomarker for sarcoidosis; however, its use has been limited by its availability [12]. Definitive diagnosis of sarcoidosis or lymphoma requires biopsy of affected tissue such as lymph nodes [9]. In our patient, a CT scan of abdomen and pelvis revealed mild retroperitoneal lymphadenopathy, with nonrevealing core biopsy. However, because core biopsy is known to have false negatives, we pursued excisional biopsy, which led to the definitive diagnosis of sarcoidosis. In cases where PTHrP and vitamin D metabolite levels are normal, further workup is warranted. This can include protein electrophoresis, bone scan, vitamin A, TSH, and morning cortisol levels to rule out potential causes such as multiple myeloma, metastatic malignancies, vitamin A toxicity, hyperthyroidism, and adrenal insufficiency [3, 4]. A detailed review of medication history is essential to exclude medication-related causes such as thiazides, lithium, as well as milk-alkali syndrome [5, 6].

The evaluation of hypercalcemia and the diagnosis of sarcoidosis were challenging in this case. Typically, pulmonary symptoms and findings of hilar or mediastinal lymph nodes are seen in 90% to 95% of patients with sarcoidosis [13, 14]. Although hypercalcemia is seen in 10% to 20% of sarcoidosis cases, isolated hypercalcemia without pulmonary involvement is rare [15, 16]. As noted previously, neither the chest radiograph nor the chest CT scan indicated any pulmonary involvement except mildly prominent retroperitoneal lymph nodes. Moreover, the normal serum ACE levels and 1,25(OH)_2_D levels during initial hospitalization, as well as a negative core needle lymph node biopsy, complicated the picture further. In a meta-analysis study, it was shown that core needle biopsies (especially using smaller gauge needles) for hilar or mediastinal lymph node sampling to assess noncaseating granulomas are associated with a false-negative rate of 10% [17]. Although a core needle biopsy of the retroperitoneal lymph node revealed benign lymphoid tissue, the excisional biopsy demonstrated the presence of nonnecrotizing granulomatous inflammation suggestive of sarcoidosis. The brisk response to glucocorticoid then mycophenolate therapy was as expected, given this pathology. This case highlights the importance of using a definitive diagnostic approach, such as excisional biopsy, in cases of non-PTH-mediated hypercalcemia accompanied by lymphadenopathy.

## Learning points

- Evaluation of non-PTH-mediated hypercalcemia requires a comprehensive workup, including medication history, assessment of PTHrP and vitamin D metabolites, imaging studies, and histopathologic examination, when indicated.
- Sarcoidosis may rarely present as isolated severe hypercalcemia in the absence of pulmonary involvement.
- In the diagnosis of sarcoidosis, core needle biopsy of lymph nodes carries approximately a 10% false-negative rate; therefore, excisional biopsy may be necessary for definitive diagnosis.

## Contributors

All authors made individual contributions to authorship. C.X., R.V., A.A., A.Y., M.Q.: design, drafting, revision, and final approval of the work. C.X., R.V., M.Q.: analysis and interpretation of data. M.Q.: diagnosis and management of this patient.

## Data Availability

Original data generated and analyzed during this study are included in this published article.
